# Actin cytoskeleton in angiogenesis

**DOI:** 10.1242/bio.058899

**Published:** 2022-11-29

**Authors:** Nidhi Yadunandanan Nair, Victor Samuel, Lariza Ramesh, Areeba Marib, Deena T. David, Ananthalakshmy Sundararaman

**Affiliations:** Cardiovascular Diseases and Diabetes Biology, Rajiv Gandhi Centre for Biotechnology, Thiruvananthapuram, Kerala, India 695014

**Keywords:** Actin, Angiogenesis, Endothelium, RhoGTPase

## Abstract

Actin, one of the most abundant intracellular proteins in mammalian cells, is a critical regulator of cell shape and polarity, migration, cell division, and transcriptional response. Angiogenesis, or the formation of new blood vessels in the body is a well-coordinated multi-step process. Endothelial cells lining the blood vessels acquire several new properties such as front–rear polarity, invasiveness, rapid proliferation and motility during angiogenesis. This is achieved by changes in the regulation of the actin cytoskeleton. Actin remodelling underlies the switch between the quiescent and angiogenic state of the endothelium. Actin forms endothelium-specific structures that support uniquely endothelial functions. Actin regulators at endothelial cell–cell junctions maintain the integrity of the blood–tissue barrier while permitting trans-endothelial leukocyte migration. This review focuses on endothelial actin structures and less-recognised actin-mediated endothelial functions. Readers are referred to other recent reviews for the well-recognised roles of actin in endothelial motility, barrier functions and leukocyte transmigration. Actin generates forces that are transmitted to the extracellular matrix resulting in vascular matrix remodelling. In this Future Leader Review, we attempt to synthesize our current understanding of the roles of actin in vascular morphogenesis. We speculate on the vascular bed specific differences in endothelial actin regulation and its role in the vast heterogeneity in endothelial morphology and function across the various tissues of our body.

## Teaser

**Summary:** Endothelial actin structures and actin-mediated force generation coordinate the multi-step angiogenic process.

## Introduction

Maintenance of a healthy vasculature is essential to nurture all tissues. Endothelial cells lining the blood vessels are not passive conduits for blood flow but actively participate in the maintenance of homeostasis in the sub-adjacent organs. Endothelial cells regulate the diffusion of oxygen, metabolites, nutrients and the passage of cells across the blood–tissue barrier. Formation of new blood vessels through vasculogenesis and further expansion of the network by angiogenesis happens through coordinated cell proliferation, migration, matrix attachment and remodelling, processes critically dependent on the actin cytoskeleton. Several recent reviews highlight the steps involved in angiogenic sprouting and the key molecular players involved in it (Eelen et al., 2020; Gurevich et al., 2021). Recent bulk and single cell RNA seq experiments reveal that indeed capillary endothelial cells exhibit enormous diversity between vascular beds with gene signatures unique to the tissues they supply (Eelen et al., 2020; Gurevich et al., 2021; Paik et al., 2020). The two non-muscle actin isoforms (β actin and γ actin) form spatially segregated structures with differences in the β/γ ratio between different cell types (Dugina et al., 2021). However, there is strong evidence that total actin levels remain the same between arteries and veins. *In vitro* as well, culture conditions like cell density and shear stress do not affect total actin content per cell but can modulate the ratio between polymerised F actin and monomeric G actin (McGrath et al., 2000; Osborn et al., 2006). Differential regulation of actin might underly the diversity in shape and function of different capillary endothelial cells. Indeed, several actin binding proteins display heterogeneity between vascular beds. Enface microscopy of endothelial monolayers in different blood vessels reveal that F-actin in veins is primarily cortical while in adult arteries the F-actin fibres run between focal adhesions oriented in the same direction as the extracellular fibronectin fibres (van Geemen et al., 2014).

Actin governs a wide array of cellular functions. However, the underlying mechanism is simple; the polymerisation of monomers into filaments and its depolymerisation back to monomers. The molecular details of general actin regulation governing its polymerisation, bundling and severing is described in several reviews (Dominguez and Holmes, 2011, Lodish, 2016). Actin cytoskeleton in the endothelium is rapidly remodelled during endothelial polarity switching and migration during angiogenesis, while also conferring stability to cell junctions to maintain barrier function. In this Future Leader Review, we will look into actin-dependent structures and actin-driven function in endothelial cells. Broadly, actin can form contractile bundles, Arp2/3 dependent branched structures as seen in lamellipodia and linear filaments as observed in filopodia. Each of these have distinct functions in invasion, migration and sensing of guidance cues in the endothelial cells, as discussed below. We will focus on uniquely endothelial functions to decipher how actin plays a central role in these processes. Finally, we will briefly explore the upstream signalling cascades that alter the actin dynamics in endothelial cells.

### Actin monomer binding proteins in angiogenesis

Profilin is a critical ATP-actin binding protein that promotes actin polymerisation. Its regulation by phosphorylation allows endothelial cells to increase actin polymerisation in response to angiogenic signalling through VEGFR2 at the leading edge of migrating cells (Fan et al., 2012). VEGF-VEGFR2 signalling is the best-studied signalling axis in angiogenesis and VEGFR2 mediated signals cause actin polarisation and cell elongation during angiogenesis (Cao et al., 2017). VEGFR2 signalling activates Src to directly phosphorylate Profilin 1 at Tyr129 in an endothelial cell-specific manner. This phosphorylation, while dispensable for developmental angiogenesis, is critical for post-ischaemic angiogenesis as shown with conditional endothelial knock-in of phsopho-deficient Pfn1(Y129F) mouse model (Fan et al., 2012). Global profilin knockout (KO) embryos die at the two-cell stage (Witke et al., 2001), while, in contrast, global KO of thymosin β4, another G-actin binding abundant protein, results in viable mice born in mendelian ratios (Banerjee et al., 2012). However, this result is refuted by another study in the same year, again with mice models, suggesting that global thymosin β4 (Tβ4)-null embryos develop haemorrhage due to reduction in smooth muscle coverage of developing vessels. This study suggests significant embryonic lethality with incomplete penetrance in Tβ4-null mice (Rossdeutsch et al., 2012). The study further demonstrates that the haemorrhagic phenotype persists when endothelial-specific Tβ4-knockdown is performed *in vivo*. Endothelium secreted Tβ4 might facilitate mural coverage of vessels. Since both papers use C56/Bl6 mice and target exon2, the difference in phenotype reported for thymosin β4 deficiency is not currently explained. A role for thymosin β4 in developmental angiogenesis is supported by the recent spatially resolved single cell RNA seq and validation experiments on chicken hearts suggesting that thymosin β4 is persistently enriched during coronary vessel development (Mantri et al., 2021). Clearly, profilin seems to play a major role in cellular actin regulation compared to thymosin β4 as the former shows a strong, completely penetrant lethal phenotype, while the latter shows incompletely penetrant phenotypes due to possible compensation by other G-actin-binding proteins.

The balancing act that maintains the equilibrium between F and G actin in cells is also through the autoregulation of actin mRNA by the monomeric G actin (Lyubimova et al., 1997). Thus, minor changes to this equilibrium contribute to cell morphological, transcriptional, and functional outputs. For example, the CTIP2 transcription factor (also known as BCL11B) is highly expressed in vascular smooth muscle cells. It mediates the phosphorylation on the actin polymerase VASP at Ser 239, inhibiting its activity. The inhibition results in a decrease in the F/G-actin ratio that modulates vascular stiffness. Indeed, a loss of CTIP2 results in the loss of the inhibitory phosphorylation on VASP, causing increased polymerisation of actin and increased aortic stiffness. The increased vascular stiffness translates to increased cerebral microbleeds in CTIP2-null mice (Valisno et al., 2021). The monomeric G actin can by itself regulate endothelial function. G actin can directly bind to the 3'UTR of endothelial nitric oxide synthetase (eNOS) mRNA (Searles et al., 2004) and can also directly interact with eNOS protein (Kondrikov et al., 2010), thereby regulating NO release and vascular tone (Fels et al., 2012). Actin also directly binds to a 27 nucleotide repeat region on the eNOS promoter regulating its expression (Ou et al., 2005). Thus, actin closely controls vascular tone by regulating the gene expression, mRNA stability and protein function of eNOS.

Actin monomers play a key role in the nuclear localisation of proteins, chromatin methylation, and transcriptional regulation (Bajusz et al., 2018). Nuclear actin also has a significant role in vascular morphogenesis. Nuclear actin is essential for the regulation of transcription. Changes in actin dynamics directly affect gene expression (Miralles and Visa, 2006). Formin-dependent actin dynamics play a crucial role in DNA replication by regulating nuclear transport and loading of replication complex proteins onto chromatin (Parisis et al., 2017). STK35L1, a nuclear Ser/Thr kinase, interacts with nuclear actin to regulate both the cell cycle and migration of endothelial cells (Goyal et al., 2011). Thus, actin itself, as well as monomeric actin binding proteins (see Table 1), plays a crucial role in maintaining an F-actin to G-actin ratio conducive to endothelial function.

**Table 1:**
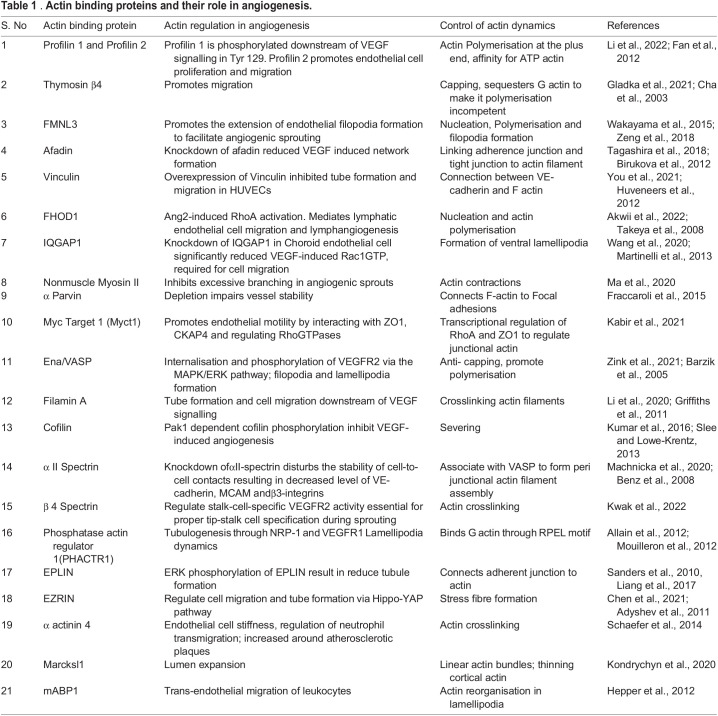
. **Actin binding proteins and their role in angiogenesis.**

### Regulators of actin polymerisation in endothelial function

An essential first step in actin polymerisation is the nucleation of a new filament. Several actin nucleators are involved in this process. These include the Arp2/3 complex with its nucleation promoting factors (NPFs), the Formin family of proteins, and tandem actin monomer-binding proteins of nucleation. Actin nucleators play several crucial roles in endothelial biology. Arp2/3 drives junctional lamellipodia formation. Inhibition of the Arp2/3 complex in endothelial cells can interfere with VE-cadherin dynamics at cell–cell junctions compromising endothelial barrier functions (Abu Taha et al., 2014). Endothelial-cell-specific postnatal Arpc4 (subunit of Arp2/3 complex) deletion results in a reduction in lamellipodial protrusions and the ability of endothelial cells to migrate into avascular areas during retinal angiogenesis, leading to severe compaction of the vascular front (Figueiredo et al., 2021 preprint). There was a striking increase in the number and length of filopodia in the endothelial cells of Arpc4-KO (inducible endothelial-cell specific KO) mice (Figueiredo et al., 2021 preprint), possibly caused by a processive lengthening of actin filaments in the absence of branching. N-WASP and WAVE proteins are the major nucleation promoting factors that regulate the Arp2/3 complex. Angiogenic signalling regulates the localisation and activity of the nucleation promoting factors. For example, in response to VEGF, there is a relocalisation of N-WASP to the cell surface through its interaction with the SH2-SH3 containing adaptor protein, Nck, which directly binds to the VEGFR2 receptor. Therefore, VEGF signalling activates nucleation of actin fibres aiding in endothelial motility (Gong et al., 2004).

Cortactin, another NPF protein, drives Arp2/3 dependent actin assembly by preventing its dissociation from the parent filament so that branch points are stabilised. Cortactin drives nucleation of actin at the leading edge of endothelial cells. As the name suggests, the protein is mainly involved in cortical actin remodelling. Like the WASP family of NPFs, cortactin is also regulated downstream of angiogenic signalling. For example, VEGF drives cortactin phosphorylation through Src kinase at the tyrosine 421 that in turn leads to its enrichment in membrane protrusions (Spring et al., 2012). Cortactin is important for endothelial migration and capillary formation in response to sphingosine-1-phosphate (Li et al., 2004).

While the nucleation promoting factors, along with Arp2/3 promote the polymerisation of actin, capping proteins generally bind to the fast-growing barbed end of actin and block the addition and loss of actin monomers. We previously discussed the relocalisation of NPFs in response to angiogenic VEGF signalling to cause actin polymerisation at specific areas. As capping proteins prevent actin polymerisation by binding to barbed end, we expect angiogenic signals to inhibit capping protein binding, to promote actin polymerisation at leading edges. For example, Hsp27, which acts as an actin capping protein, is indeed sensitive to VEGF. Phosphorylation of Hsp27 leads to an inhibition of the actin filament capping and only the non-phosphorylated monomers of Hsp27 can cap actin filaments (Landry and Huot, 1999). In response to VEGF signalling, the SAPK2 kinase phosphorylates and therefore inhibits Hsp27 dependent capping, promoting actin reorganisation (Landry and Huot, 1999). Similarly, MK2-dependent phosphorylation of Hsp27 promotes actin remodelling downstream of TNFα signalling (Chang et al., 2011).

Similarly, actin filament capping by tropomodulin (Tmod3) causes a reduction in cell motility in endothelial cells, presumably by preventing lamellipodial protrusions that require actin branching. Interestingly, Tmod3 is unique in being able to cap the pointed end of actin fibres. Tmod3 dependent pointed end capping significantly reduces F-actin in the lamellipodia while G actin remained unaltered. Also, the pointed end capping by overexpressed Tmod3 reduces the number of free barbed ends available in the cell. Capping at the pointed end by Tmod3 is then expected to give rise to longer-lived filaments due to the slow rate of depolymerisation. These longer-lived filaments with more ADP-Actin might not support Arp2/3-dependent branching as Arp2/3 nucleates from ATP actin subunits. Decreased branching will reduce the number of barbed ends available and thus explains the reduction in F-actin at the lamellipodia (Fischer et al., 2003).

Formins are another class of proteins that control the nucleation and polymerisation of new actin filaments. Formins contain the FH1 domain with polyproline tracks that bind to profilins for the processive elongation of actin filaments (Paul and Pollard, 2008). Most formins also contain a GTPase-binding domain (GBD). RhoGTPases activate formins by disrupting an intramolecular inhibitory interaction (Watanabe et al., 1997). As proteins aiding in processive filament elongation, formins are involved in filopodia formation, assembly of stress fibres, actin networks for vesicular transport, cell adhesion, and stabilisation of endothelial junctions (Schonichen and Geyer, 2010).

While the physical binding of the active RhoGTPase to the GBD disrupts the inhibitory interaction between FH3 and DAD domains in formins, phosphorylation events can also regulate the inhibitory state of formins. For example, the phosphorylation of the formin FHOD1 by RhoA/ROCK releases FHOD1 from its autoinhibited state and induces stress fibre formation (Takeya et al., 2008). Differential RhoGTPase activation downstream of angiogenic signals would correspondingly activate formins to nucleate and form long actin filaments. For example, mDia activation by RhoA downstream of Angiopoietin1 signalling promotes endothelial junctional stability (Gavard et al., 2008).

Filopodial protrusions in the direction of the pro-angiogenic signal helps in efficient endothelial cell migration during angiogenesis (Mellor, 2010). In an siRNA screen for formins playing a role in angiogenesis using an *in vitro* co-culture assay model, FMNL3, DAAM2, and FHOD1 were found to be critical regulators (Yamada and Nelson, 2007). FMNL3 is a regulator of endothelial cell elongation during angiogenesis. Overexpressing FMNL3 in endothelial cells causes an intense reorganisation of actin. FMNL3 acts downstream of RhoJ to mediate the polarised trafficking of podocalyxin to the endothelial AMIS, initiating vascular lumen formation (Richards et al., 2015). FMNL3 also helps in maintaining a pool of dynamic F-actin filaments at endothelial cell junctions that reinforces junction stability, thereby aiding lumenogenesis (Phng et al., 2015). Regulators of filopodial extensions is dealt with in detail in subsequent sections. Key regulators of actin nucleation and polymerisation are summarised in table 1

## Endothelial actin structures

### Junction associated lamellipodia

JAILs are small (1-5 μm) actin-driven protrusions that form at adherens junctions. They regulate VE-cadherin dynamics at cell–cell junctions (Cao and Schnittler, 2019; Cao et al., 2017). JAILs are formed in response to VEGF signalling induced cell elongation that leads to localised reduction in VE-cadherin concentrations. This triggers Arp2/3 and Rac dependent actin polymerisation leading to junctional small JAILs. JAILs have been demonstrated *in vivo* in developing mouse retinal vessels at the tip-cell–stalk-cell interface (Cao et al., 2017). Indeed, localised MLCII dephosphorylation downstream of VEGFR2 signalling can reduce membrane tension locally to permit JAIL formation (Cao et al., 2017). In endothelial cell migration, junctional oscillations play a crucial role and these junctional protrusions have been confirmed *in vivo* in zebrafish (Paatero et al., 2018). They are variously called junction associated intermittent lamellipodia (JAILs) or junction based lamellipodia (JBL). JAILs causing overlap between adjacent endothelial cells was initially described in (Abu Taha et al., 2014). The interactions in trans create new VE-cadherin contact sites. JAILs play a crucial role in endothelial cell–cell communications (Cao and Schnittler, 2019). The relevance of JAIL in regulating junctional permeability is evident in the transient inhibition of JAIL formation in response to thrombin which increases endothelial permeability (Cao and Schnittler, 2019).

JBL forms when two tip cells come together during anastomosis and lumen formation. The role of JBL was explored during angiogenesis in zebrafish, where these structures promoted junctional elongation and cell–cell rearrangements (Paatero et al., 2018). When two tip cells come together for anastomosing, the F-actin protrudes from a stable distal junction. VE-cadherin then diffuses onto the distal end, establishing inter-endothelial contacts. The new contacts serve as a clutch, and the F-actin remodelling provides the motive force that pulls the proximal junction towards the newly formed distal junction. Hence, the endothelial cells move onto one other via VE-cadherins (Paatero et al., 2018).

While JAILs and JBLs are recently described junctional actin structures, several follow-up studies describe key molecular players in this process. Signalling through the YAP/TAZ pathway causes increased VE-cadherin turnover and JAIL formation. JAIL helps endothelial cells maintain the barrier function while undergoing cell rearrangements during angiogenesis (Neto et al., 2018). Stretch-induced mechanical signals are integrated with BMP signalling by the YAP/TAZ pathway to regulate JAILs. Interestingly, loss of this pathway causes an increase in F-actin bundles and a loss of branched actin networks that characterise the JAIL structure. Thus, a lack of YAP/TAZ leads to straighter junctions with less VE-cadherin turnover (Neto et al., 2018).

Actin binding proteins like coronin 1B (Werner et al., 2020), α-Parvin (Fraccaroli et al., 2015), and EPLIN α and β (Taha et al., 2019) are critical regulators of JAIL formation (Seebach et al., 2020). In summary, JAILs and JBL are under the control of mechanical cues like shear stress or stretch. They depend on branched actin formation under the control of Arp2/3, much like the classical lamellipodia, and are driven by Rac1, WASP, and WAVE family proteins. Junctional actin structures are critical regulators of VE-cadherin-mediated endothelial barrier properties.

Membrane blebbing in endothelial cells is also actin dependent. While there are very few studies of membrane blebbing on endothelial cells, it has been described in response to toxic stimuli like peroxide and ouabain (Özdemir et al., 2015; van Gorp et al., 1999). It is primarily driven by RhoA-ROCK mediated cortical actin contraction. Blebbing in endothelial cells can be induced by diabetic retinopathy and in this context, in human samples, ROCK was demonstrated in endoluminal blebs (Rothschild et al., 2017). While membrane blebbing is associated with cell death, it could also play a physiological role in cell spreading and precedes the appearance of lamellae and delays its appearance in spreading endothelial cells (Norman et al., 2011). Blebs differ from JAILs in their involvement of cortical actin and the ROCK pathway instead of Rac1 and junctional actin. Crucially both blebbing and JAILs dependent on a reduction in junctional VE-cadherin density (Corada et al., 1999).

### Apical Membrane Insertion Site (AIMS)

F-actin localises in the form of a bleb or a bud on the apical side of the cell from which long actin cables radiate, carrying prospective cargo to the early apical surface (Richards et al., 2015). This structure is designated as the AMIS in endothelial cells (Richards et al., 2015). Linear actin cables are nucleated from the AMIS. FMNL3 increases the size of the AMIS, suggesting that it is the actin nucleator at AMIS. FMNL3 coordinates the delivery and incorporation of podocalyxin via F-actin cables into the apical surface leading to the formation of a mature lumen (Richards et al., 2015). AMIS is rich in Ezrin/Rodixin/Moesin (ERM)-family proteins, which physically interact with podocalyxin and actin proteins and help in lumenogenesis. Cell polarity is established through coordinated regulation of both the actin and microtubule cytoskeleton. FMNL3 also regulates endothelial cell microtubules during angiogenesis (Hetheridge et al., 2012). FMNL3 interacts with RhoGTPases Cdc42 and RhoJ, and they act as upstream activators of FMNL3.

VE-cadherin and Moesin are also involved in establishing a polarised apical membrane surface. Moesin positions the F-actin-rich bud correctly at the apical surface (Galvagni et al., 2012). *In vivo* studies also show that F-actin localises to the juxtaposed early apical surface between endothelial cells and VE-cadherin is a pre-requisite for Moesin and F-actin recruitment. Finally, F-actin-Myosin-II-mediated contractility opens up and stabilises the nascent extracellular lumen. Interestingly, here podocalyxin and ERM proteins like Moesin are reported to be essential for F-actin recruitment to the apical membrane (Strilić et al., 2009). The exact order of protein recruitment to the early apical surface that facilitates lumenogenesis remains controversial.

### Kugeln

‘Kugeln’ is a German term that translates to ‘sphere’. It refers to round protrusions formed on the endothelial membrane in transgenic zebrafish with an actin-rich neck region. They are nitric oxide containing structures that are found exclusively on cerebral vessels in zebrafish (Kugler et al., 2019). They do not communicate with the vessel lumen, form in the absence of flow, and contain little to no cytoplasm. These structures are actin and Notch-dependent, and their formation is inhibited by VEGF and Wnt signalling (Kugler et al., 2019). Their functional significance remains a mystery.

### Podosomes

Podosomes refer to specialised cell-matrix contact sites capable of extracellular matrix degradation. They are characterised by the presence of F actin, cortactin, and metalloproteinases. Cultured endothelial cells often form 5–10-µm-wide, ring-like clusters of podosomes called rosettes. The podosome-mediated degradation of the vascular basement membrane is critical for sprouting angiogenesis (Seano et al., 2014b). These structures have an actin core formed by actin nucleators (Arp2/3), actin binders (tropomyosin), polymerisation activators (WASP), and GTPases (RhoA, Cdc42, dynamin) as well as a ring formed by adhesion molecules like vinculin (Gimona et al., 2008; Moreau et al., 2003).

Podosome formation in endothelial cells occurs through the initial mobilisation of the scaffolding protein Tks5 and F-actin accumulation. Subsequently, Septin 2 gets recruited, which regulates podosome maturation (Collins et al., 2020). Septin 6, septin 7, and septin 9 are localised to Src-induced podosome formation (Collins et al., 2020). GIT1 is an important mediator of VEGF-induced podosome formation in endothelial cells (Wang et al., 2009). VEGF-dependent upregulation of α6β1 and its subsequent translocation into podosomes is critical for degradation of the matrix and formation of branch points on the vessel during angiogenesis (Seano et al., 2014a). *Ex vivo* cultured aortic vessel segments show endothelial podosome rosette formation in response to TGF β (Varon et al., 2006). Podosomes degrade the underlying basement membrane proteins like collagen IV, thereby playing a role in arterial vascular remodelling (Rottiers et al., 2009). Tip cells assemble podosomes during physiological angiogenesis *in vivo* (Spuul et al., 2016). While VEGFA promotes the formation of podosome rosettes with high matrix degradation potential, the Notch ligand Dll4 negatively regulates podosomes (Spuul et al., 2016). ARHGAP35 (p190A RhoGAP) negatively regulates the number of podosome structures in endothelial cells, while the closely related p190B RhoGAP (ARHGAP5) promotes the localisation of MT1-MMP and MMP2 in podosome structures regulating their ability to degrade the ECM locally without altering podosome numbers (Guegan et al., 2008). Interestingly, both p190ARHGAPs act on RhoA primarily (Muller et al., 2020), suggesting that their isoform-specific function in endothelial cells is perhaps independent of their action on RhoA. Protein kinase A (PKA) is yet another negative regulator of podosome rosettes in endothelial cells through the inhibition of Cdc42 activity by increasing Cdc42 interaction with RhoGDIα (MacKeil et al., 2019). Taken together, the formation and maturation of podosomes are tightly regulated by actin remodelling proteins and their upstream angiogenic signals to ensure stepwise progression of new vessel formation.

### Filopodia

Filopodia have long parallel actin filaments arranged in a tight bundle. They are finger-like projections with the fast-growing barbed ends of actin filaments orientated towards the plasma membrane (Mallavarapu and Mitchison, 1999). Filopodia are classically thought of as mediating directional cell migration in response to extracellular cues. In the context of angiogenesis, they serve to sense the gradients of growth factors like VEGF-A, particularly in tip cells as indicated in mice retinal angiogenesis models (Gerhardt et al., 2003). However, later studies from the Gerhardt group using zebrafish models expressing Life-act EGFP in endothelial cells demonstrated that an inhibition of filopodial protrusion with low doses of Latrunculin B does not inhibit directional migration of intersegmental vessel endothelial cells. Lamellipodia provide sufficient driving force for migration and the directionality is fully compensated for by the lamellipodial protrusions in the developmental angiogenesis of intersegmental vessels (later study from Gerhardt group). Interestingly, tip-cell specification and ectopic branching in the absence of Notch ligand Dll4 were not dependent on filopodia but vessel anastomosis did depend on endothelial cell filopodia (Phng et al., 2013).

More recently, it was demonstrated that specialised broader membrane ruffles arising from filopodia called dactylopodia are used by tip cells for invasion into the extracellular matrix. The balance between Arp2/3 and Myosin IIA (NMIIA) activity determines the number of filopodial and dactylopodial protrusions (Figueiredo et al., 2021). While these structures are functionally similar to podosomes in their ability to promote cell invasion, dactylopodia are formed through a conversion of pre-existing filopodia through Arp2/3-dependent branching while Arp2/3-deficient endothelium ends up forming excessive filopodia without dactylopodial maturation. Indeed, the role of NMIIA in keeping Arp2/3 activity in check to promote filopodial stability *in vivo* suggests that the regulation of dactylopodia is different from that of podosomes. Podosomes can form rosettes in sprouting vessels *in vivo* and the process is negatively regulated by Notch signalling. Indeed, inhibition of Notch can lead to podosomes in stable ensheathed vessels of the retina as well (Spuul et al., 2016), while dactlopodia seem to be highly tip-cell specific.

During endothelial sprouting, the tip cells extend multiple filopodia, which are enriched in VEGF receptors, particularly VEGFR2/3 heterodimers (Nilsson et al., 2010), which enable the cells to sense gradients of VEGFA and VEGFC. Cdc42 is the key RhoGTPase linked to filopodia formation. For example, BMP signalling, through the Cdc42-FMNL3 axis, regulates filopodia formation in endothelial cells (Wakayama et al., 2015). Bradykinin, an inflammatory mediator, induces the formation of filopodia via the Cdc42 pathway (Petrovic et al., 2007). FGD5 is an EC-specific RhoGEF that binds and activates Cdc42 (Cheng et al., 2012). Not surprisingly, FGD5 is also a key regulator of endothelial filopodial number and length (Farhan et al., 2017). FGD5 promotes vascular pruning in mouse retinal model of angiogenesis (Cheng et al., 2012). YAP/TAZ transcription factors regulate actin remodelling at filopodia and cell–cell junctions. Endothelial-specific deletion of YAP/TAZ leads to fewer filopodia and disarranged tight as well as adherens junctions (Kim et al., 2017). Lats1/3 inducible deletion in endothelial cells is a tool used by these authors to hyperactivate the YAP/TAZ pathway. This showed several phenotypes like hypernumeric filopdial extensions from the tip cells and hyperproliferation of endothelial cells. Loss of YAP/TAZ reduces VEGF-induced Cdc42 activity but not RhoA and Rac1. The loss of Cdc42 activity was traced to a reduction in total protein levels of Cdc42. This leads to a loss of MLC2 activity that possibly controls junctional integrity and filopodial protrusions in endothelial cells (Kim et al., 2017). Alongside Cdc42, several actin cytoskeletal regulators are downregulated in YAP/TAZ deficient brain endothelial cells compared to WT, demonstrating a multi-faceted regulation of the actin cytoskeleton by the YAP/TAZ pathway.

**Fig. 1.**
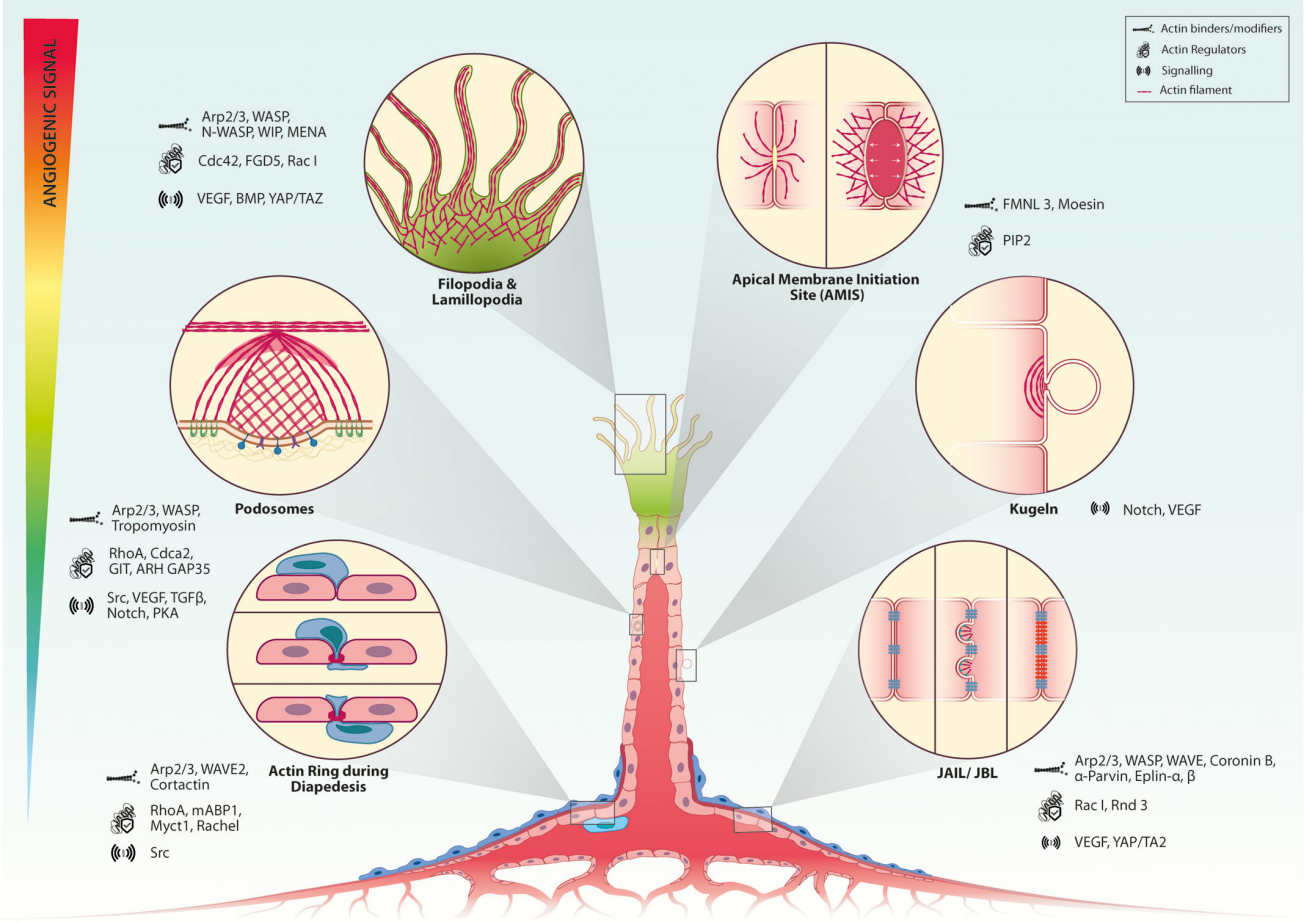
**Actin structures and dynamics in angiogenesis**. Key signalling pathways and effectors are shown without being exhaustive.

RhoA is also implicated in filopodia formation downstream of angiogenic signals. Syx, a RhoA GTPase exchange factor (GEF), serves as an important protein that binds with angiomotin (Amot) and regulates the activity of RhoA (Ernkvist et al., 2009). Filopodia contact the ECM to initiate the focal contact points through focal adhesion kinase (FAK) (Mitra et al., 2005). Filopodia are hence the first contact between the ECM and migrating cells and help in cell spreading through small Rho GTPases. The Slit-Robo pathway promotes filopodia formation in endothelial cells through the direct interaction of Robo4 with actin nucleators: WASP, N-WASP, WIP, and Mena (Sheldon et al., 2009). Endothelial actin structures are depicted in figure 1 along with the key actin regulators and signalling pathways that regulate them.

## Mechano-responsive actin regulators in endothelial cells

The YAP/TAZ pathway is indeed one of the key mechano-sensitive pathways with multifaceted roles in actin remodelling. Hemodynamic shear stress-mediated lumen stabilisation is dependent on actin cytoskeletal remodelling. Shear stress causes a thickening of cortical actin bundles in endothelial cells that recruit the AMOT protein. The AMOT-family proteins are negative regulators of the YAP/TAZ signalling (Zhao et al., 2011). AMOT recruitment to cortical actin disrupts the AMOT-YAP inhibitory complex in the cell cytoplasm. This leaves YAP free to move into the nucleus and trigger transcriptional responses critical for the maintenance of vascular lumen *in vivo* (Nakajima et al., 2017).

Yet another AMOT/Motin family member, Angiomotin-like 2 (AMOTL2), acts as a scaffold protein linking VE-cadherin to contractile actin fibres. This function was shown to be critical for aortic lumen formation and expansion in zebrafish (Hultin et al., 2014). The function of AMOTL2 in linking VE-cadherin to MAGI1 bound actin filaments suggests that force transmission between neighbouring cells is critical for lumen expansion (Hultin et al., 2014). Upon AMOTL2 KO, the polarised endothelial cells lack radial actin filaments causing a collapse of the lumen and failed vessel morphogenesis (Hultin et al., 2014).

Another endothelial mechanosensor, Piezo1, responds to shear stress and sphingosine-1-phosphate through an increased calcium-gating function. This plays a crucial role in lumen formation in response to hemodynamic shear stress (Kang et al., 2019). Piezo1 facilitates F-actin alignment along the direction of flow (Ranade et al., 2014). Additionally, Piezo1 ion gating is sensitive to actin cytoskeletal and the lipid bilayer tension (Wang et al., 2022). Marcksl1 is yet another recently described mechanoresponsive actin regulator in endothelial cells (Kondrychyn et al., 2020). Marcksl1 directly binds to actin to regulate cell shape. Marcksl1 promotes the formation of linear actin bundles while reducing the actin at the cell cortex, making the cell more pliable in response to shear stress. Lumen expansion requires thinning of apical cortical actin, and Marcksl1 overexpression leads to increased vessel diameters (Kondrychyn et al., 2020). As a corollary, the loss of Marcksl1 causes delays in the luminisation of vessels in zebrafish. While Marcksl1 predominantly affects cell shape, endothelial cell numbers also determine the size of the lumen. Modulators of endothelial cell proliferation increase luminal diameter, like the protein intermidin. Intermidin forms a complex with Src and β arrestin1 to activate ERK signalling and endothelial cell proliferation to cause lumen enlargement (Wang et al., 2018). Whether the effect of intermidin on endothelial cell proliferation is dependent on its ability to inactivate the RhoA–ROCK pathway (Aslam et al., 2011; Aslam et al., 2012) needs to be investigated.

Vascular endothelial protein tyrosine phosphatase (VE-PTP/PTP-RB) is an endothelium-enriched gene that dephosphorylates substrates present in the endothelial cell junctions such as Tie-2, VE-cadherin, and plakoglobin (Dominguez et al., 2007b; Winderlich et al., 2009). VE-PTP has also been found to associate with and dephosphorylate VEGFR2 (Mellberg et al., 2009). Studies reveal that shear-induced stress in endothelial cells leads to the polarised distribution of VE-PTP at the downstream edge of cells relative to the direction of flow (Mantilidewi et al., 2014). Shear-stress-induced VE-PTP distribution and internalisation is disrupted by inhibition of actin polymerisation and Cdc42 activity, indicating that VE-PTP activity in endothelial cells requires the reorganisation of the actin cytoskeleton (Mantilidewi et al., 2014). VE-PTP deletion results in embryonic lethality due to a failure to form branched vascular networks (Dominguez et al., 2007a).

## Endothelial actin in leukocyte diapedesis

Trans-endothelial migration (TEM) refers to immune cells crossing the endothelial barrier during inflammation and immune surveillance. Leukocyte recruitment involves initial rolling, arrest and finally transmigration. The leucocytes migrate either directly through the individual microvascular endothelial cells (transcellular route) or between adjacent cells (paracellular route). Leucocytes form invasive podosomes to establish a transcellular pore in endothelial cells. The leukocytes initially extend a filopodia-like structure into the endothelial membrane or their paracellular junctions during diapedesis. This structure elongates to form a leading edge in the migrating cell and is termed the ‘pseudopodium’. Rac1, Rac2, and Cdc42 stabilise these pseudopodia for pore generation during TEM (Heasman and Ridley, 2008). The pores are maintained by F-actin-rich rings that are formed around squashed leucocytes during diapedesis. The actin rings are regulated by RhoA (Heemskerk et al., 2016).

During paracellular transmigration, many adhesive interactions, including the JAM-A, PECAM-1, and CD99, are exploited by the leukocytes (Sullivan and Muller, 2014). Moreover, the VE-cadherin/α catenin must be disassembled from the actin cytoskeleton for the dissolution of adherent junctions of endothelial cells (Vestweber, 2012).The attachment of leucocytes to endothelial cells requires WAVE2- and Arp2/3-driven actin polymerisation, which creates a docking structure (Mooren et al., 2014). WAVE2 localises to the transcellular pore, and its knockdown reduces transcellular migration of leucocytes with no effect on paracellular migration (Mooren et al., 2014). Thus, distinct endothelial actin modulators govern the route of leucocyte diapedesis.

Endothelial intercellular adhesion molecule (ICAM1) is a key regulator of leukocyte transmigration as it interacts directly with leukocyte β2 integrins. ICAM1 clustering enables binding to leucocyte integrins, facilitating leucocyte adhesion. ICAM1 clusters recruit β actin and filamin. Cortactin dynamically links ICAM to actin and studies demonstrate that the tyrosine phosphorylation of cortactin by Src is essential for actin remodelling. Indeed, knockdown of cortactin in endothelial cells reduces actin-ICAM clustering around adherent PMNs (Yang et al., 2006). Clustering of ICAM also results in the rise in the concentration of intracellular calcium (Ca^2+^) and p38 mitogen-activated protein kinase (MAPK) (Hu et al., 2000). Actin polymerisation is critical for further ICAM1 recruitment and growth of clusters. Actin-dependent inside-out regulation of ICAM1 in the endothelium is therefore essential for leucocyte transmigration (van Buul et al., 2010). Together, ICAM1 dependent coupling of actin to the TEM site feeds forward to allow the completion of TEM without significant barrier disruption.

Mammalian actin binding protein 1 (mABP1) also plays a specific role in trans-endothelial leucocyte migration under shear stress. The protein is enriched in the leading edge and causes dynamic actin rearrangements in the lamellipodia. Furthermore, the binding of mABP1 to actin is modulated by β2 integrin driven leucocyte adhesion to endothelium, suggesting that actin remodelling by partners with changing binding affinities could coordinate the multistep process of leucocyte diapedesis (Hepper et al., 2012).

Another gene with near exclusive endothelial cell expression, Myct1, was shown to be critical in the context of tumour angiogenesis where targeting Myct1 showed promises in the tumour vessel normalisation. This is because Myct1 deficient endothelial cells facilitate trans-endothelial migration of cytotoxic T lymphocytes. This increased T cell migration phenotype upon Myct1 depletion is abolished by pharmacological Rac1 inhibition. Myct1 deficient endothelial cells have increased expression of Rac1 associated effectors like Trio and Tiam alongside increased expression of adhesion molecules like E selectin, P selectin, ICAM and VCAM (at mRNA level). Together, this suggests that increased junctional Rac1 activation could facilitate TEM of immune cells (Kabir et al., 2021). Since Rac1 is indeed a key player in the junctional lamellipodia (JBL and JAIL) formation, its hyperactivation could lead to more fluid membrane dynamics and consequently less resistance for cell migration across endothelial cells. Since Rac1 deficient endothelial cells show hemorrhagic manifestations, Rac1 is essential for embryonic vascular integrity (Nohata et al., 2016; see also table 2). Trans-endothelial migration of carcinoma cells was previously demonstrated to be Rac1 dependent with Rac1 knockdown or single copy deletion *in vivo* attenuating carcinoma cell trans-endothelial migration and hematogenous spread. Conversely, VEGF-induced Rac1 activation supported trans-endothelial migration of tumour cells (Yao et al., 2015). Indeed, Rac1 activation at junctions seems to be conducive to trans-endothelial cell migration of both tumour cells and lymphocytes.

**Table 2.**
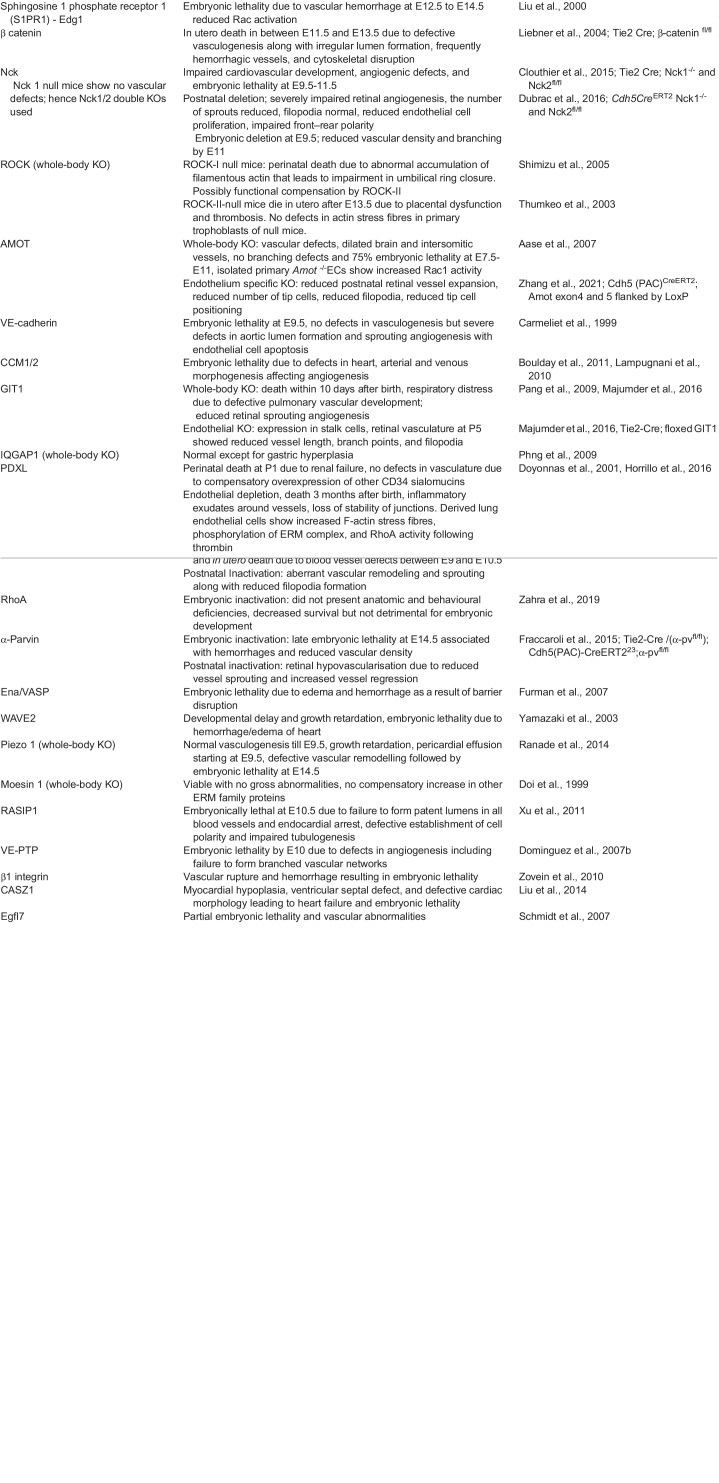
Angiogenic phenotypes of mice models of actin regulator deletions.

## Actin in endothelial to mesenchymal transition (EndMT)

EndMT is a cellular transdifferentiation process similar to the epithelial to mesenchymal transition (EMT), which occurs in both developmental and pathological settings. Endothelial cells lose their cobblestone morphology during this transition by reducing many junctional proteins like CD31 (PECAM), ZO1, and VE-cadherin. A switch in polarisation ensues and results in the acquisition of the classical mesenchymal markers like vimentin, N cadherin, and α smooth muscle actin (αSMA). EndMT has a role in the embryonic development of cardiac valves and in human diseases like malignancies, fibrotic diseases, pulmonary arterial hypertension, atherosclerosis, and diabetes mellitus (Bischoff, 2019). The remarkable change in cell shape and acquisition of motility is accompanied by actin cytoskeletal reorganisation.

**Fig. 2:**
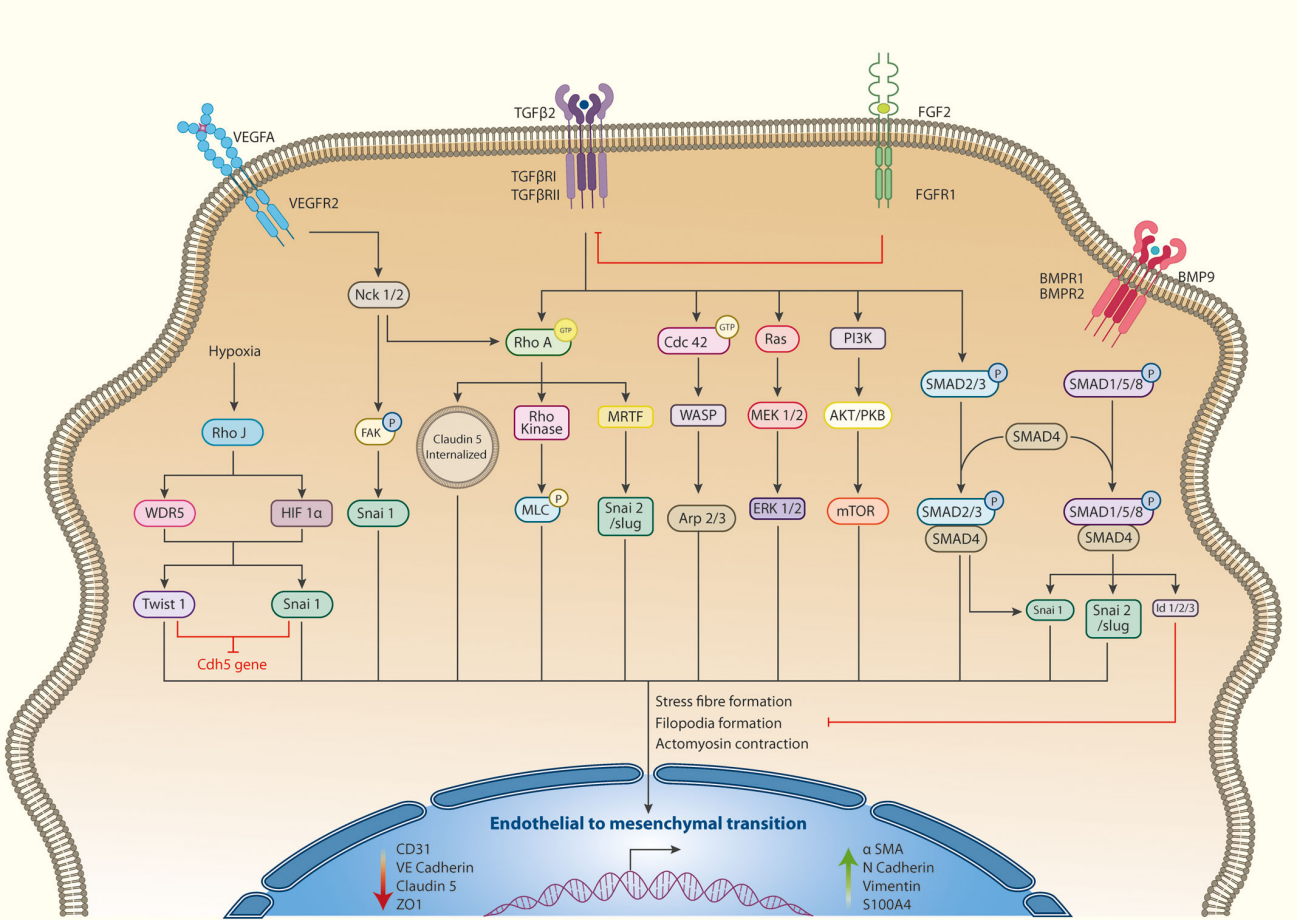
Signalling pathways regulating actin in endothelial to mesenchymal transition.

Actin dynamics in EndMT are initiated downstream of the classical TGFβ signalling pathway. While TGFβ through Smad2/4 upregulates EndMT transcription factors like Snail, Slug, Twist, and MRTF-A, the signalling pathway activates RhoGTPases RhoA, RhoB, Rac1, and Cdc42 in a Smad-independent manner to orchestrate cytoskeletal reorganisation (Ciszewski et al., 2017). The adapter protein Nck is also a critical actin regulator in the context of EndMT as well. During the development of the heart valve, cardiac cushions are formed through EndMT of a subset of endothelial cells (Armstrong and Bischoff, 2004). In mice with endothelium-specific Nck KO, this transition is delayed, suggesting that Nck is required for EndMT *in vivo* (Clouthier et al., 2015). Cells lacking Nck protein display reduced RhoA and increased Rac1 activity, resulting in altered actin organisation with defects in cell migration (Clouthier et al., 2015). Thus Nck, TGFβ, canonical Wnt, and Notch signals promote EndMT, while FGFR1 and BMPR2 signals often counteract TGFβ to suppress EndMT Figure 2. Readers are referred to reviews (Souilhol et al., 2018; Lin et al., 2012; Piera-Velazquez and Jimenez, 2019) for details of pathways driving EndMT.

EndMT occurs in various pathophysiological conditions in response to inflammation, shear stress, cyclical strain, and oxidative stress (Krenning et al., 2016). Several of these upstream cues also modulate Rho GTPase activity, and generally, we find an increase in RhoA coupled to a reduction in Rac1 activity favouring EndMT. However, a few studies also propose a reduction in RhoA activity during EndMT. TGFβ induced miRNAs miR-27 and miR-31 target Vav3, a GEF for RhoA (Muller et al., 2020), promoting EndMT (Katsura et al., 2016; Suzuki et al., 2017). Since the bulk of scientific evidence supports RhoA as the central effector of EndMT, the miRNA-mediated Vav3 targeting should be revisited in this context.

RhoJ activates EndMT downstream of hypoxia through transcriptional activation of EMT transcription factors like Snail and Twist (Liu et al., 2018). However, how hypoxia causes RhoJ activation and RhoJ-dependent upregulation of histone H3K4 methyl transferase WDR5 to promote EndMT remains a mystery. Surprisingly, this study does not address the role of RhoJ in coordinating actin reorganisation during EndMT, a likely avenue of research in the future.

## Actin in endothelial phagocytosis-like processes

Endothelial phagocytosis is a surveillance strategy to remove foreign particles from the blood. In Listeria infection, endothelial cell behaves as a non-specialised phagocyte, with Listeria adhesion and phagocytosis-like uptake process. PI3K/FAK/Rho and FMNL3 are the key players of endothelial phagocytosis-like processes. Listeria monocytogenes exploits the endothelial phagocytosis-like process for entry, using its Act A protein as an Arp2/3 activator. The bacterium then uses actin comet tails to move freely in the endothelial cell cytoplasm. RhoGTPase effector kinase (ROCK) helps in the adhesion of Listeria onto the HUVEC. The internalisation is controlled by the combined action of the Formin family of nucleators (FHOD1 and FMNL3) and actin polymerisation proteins (Nck 1, DLG1, Rac2, PFN1, and MYO9A), and consequently need less cellular Arp 2/3 activity (Rengarajan et al., 2016). Endothelial cells are also known to internalise/phagocytose senescent neutrophils, apoptotic debris, and neutrophil extracellular traps (NETs). If NETs are not promptly cleared by endothelial phagocytosis, they promote vascular leakiness and EndMT (Pieterse et al., 2017).

## Actin in vascular matrix remodelling

Endothelial cells along with the supporting cells assemble a laminin rich basement membrane in quiescent vessels. This basement membrane is degraded during angiogenic invasion using matrix metalloproteinases (Davis and Senger, 2005). Indeed, the angiogenic ECM is rich in fibronectin and serves as a mechanical support to organise the sprout. Additionally, the angiogenic ECM determines growth factor availability and influences the gradient of growth factors available to tip and stalk cells. ECM signals through integrins to regulate cell behaviour and polarity. As is well appreciated in other tissues, endothelial remodelling of the ECM is dependent on actin cytoskeletal tension exerted on the ECM proteins through integrins to unfurl and cause the crosslinking of matrix. For example, endothelial fibronectin remodelling is regulated by the trafficking of active α5β1 integrins. The antagonistic action of the two RhoGTPases, RhoJ and Cdc42, govern the trafficking of integrins to finetune fibrillogenesis at the angiogenic front (Sundararaman et al., 2020; Sundararaman and Mellor, 2020). Collagen I instructs endothelial morphogenesis by promoting actin polymerisation into stress fibres. This is specific to dermal endothelial cells and achieved by ligation of Collagen I to integrin receptors with subsequent suppression of PKA activity (Whelan and Senger, 2003). Together, we have actin regulation of matrix bundling and matrix molecules in turn altering actin cytoskeletal organisation for vascular morphogenesis.

## Role of actin regulators in developmental and postnatal angiogenesis - lessons from global and conditional KO mice models

Several actin regulators are critical for vasculogenesis and early angiogenesis, leading to embryonic lethality in KO animals. We presently have a more nuanced understanding of the role of actin cytoskeletal regulators through endothelial cell specific KO animal models where the deletion is induced in late embryonic and postnatal mice. A compilation of the phenotypes of actin regulators; whole body, and conditional endothelial cell KOs in mice are given in Table 2.

## Conclusions and future directions

In this synthesis of the literature, we provide a conceptual framework to understand actin regulation in endothelial cells. The RhoGTPase family of proteins, particularly RhoA, Rac1, and Cdc42, play crucial roles in coordinating actin remodelling during angiogenesis. We are now beginning to realise the role of less-studied members of this family like RhoB (Gerald et al., 2013; Wheeler and Ridley, 2004), RhoC (Del Galdo et al., 2013), RhoG (Abraham et al., 2015), and RhoJ (Fukushima et al., 2020; Sundararaman and Mellor, 2020; Kim et al., 2014; Kusuhara et al., 2012; Leszczynska et al., 2011) in angiogenesis. We are only beginning to understand the role of crosstalk between RhoGTPase family members and their competition for common substrates/effectors in the process of angiogenesis (Sundararaman and Mellor, 2020). In other systems, we are aware of extensive crosstalk between RhoGTPase family members to achieve spatially restricted RhoGTPase activation (Muller et al., 2020). This is only compounded by the presence of several shared regulators - the GEFs and GAPs (Muller et al., 2020) and GDI proteins (Garcia-Mata et al., 2011). Rho GTPases lacking GTPase activity, like the Rnd subfamily and RhoH, inhibit the RhoA and Rac GTPases respectively and play crucial roles in fine-tuning their activity in other systems (Li et al., 2002; Tajadura-Ortega et al., 2018; Wennerberg et al., 2003). There is a need to explore the crosstalk between the RhoGTPase family members in angiogenesis to define the spatial and temporal regulation of actin dynamics at various stages of angiogenesis.

Pro-angiogenic and anti-angiogenic stabilising signals are activated in a temporally coordinated fashion to enable the sprouting and luminisation of new vessels. VEGF and Notch signalling, Semaphorin-PlexinD1 signalling, Slit-Robo pathways, and the mechano-sensitive YAP/TAZ pathways; all play key roles in coordinating the multi-step angiogenesis program. Actin dynamics is the end-point effector for all these pathways to bring about endothelial shape changes and vascular morphogenesis.

It is essential to look closely at endothelium-enriched actin regulators, particularly those that have arisen in the vertebrates with the advent of the closed-blood vascular system. Proteins like ARHGEF15 and its substrate RhoJ (Takase et al., 2012) fine-tune actin remodelling by more ancestral regulators in endothelial cells during angiogenesis. These proteins could be a key to understanding how actin regulation is tailored to suit the unique roles of the endothelium in our body. Actin dynamics is at the crux of endothelial plasticity. Recently discovered membrane protrusions like JAIL and dactylopodia needs to be further investigated in physiological and pathological angiogenesis. With the advent of single-cell RNAseq and spatial transcriptomic studies, we are unearthing endothelial cell fate transitions inherent in the development and maintenance of vasculature. With the phenotypic specialisation of the endothelium in distinct organs, a corresponding nuanced modulation of actin to give rise to distinct actin-based structures is expected, but the molecular details remain unknown, warranting further study.
